# Structural Analysis of Valine Residues in Silk Fibroin by Solid-State NMR

**DOI:** 10.1021/jacs.5c13210

**Published:** 2025-09-25

**Authors:** Misaki Mizushima, Takashi Mizuno, Mitsuru Toda, Mike P. Williamson, Yu Suzuki

**Affiliations:** † Department of Applied Chemistry and Biotechnology, Graduate School of Engineering, 26423 University of Fukui, 3-9-1, Bunkyo, Fukui-shi, Fukui 910-8507, Japan; ‡ 119855 JEOL ltd., 3-1-2, Musashino, Akishima-shi, Tokyo 196-8558, Japan; § School of Biosciences, 7315 University of Sheffield, Firth Court, Western Bank, Sheffield S10 2TN, U.K.

## Abstract

The silk fibroin produced by the silkworm *Bombyx mori* has a highly repetitive sequence, composed mainly of repeats of GAGAGS or GAGAGA, with some of the A/S residues being replaced by Y or V. Silk fiber is approximately 60% composed of crystalline regions, separated by less structured regions, generally described as amorphous. The crystalline regions are widely thought to be composed mainly of GAGAGS and GAGAGA repeats, and the role of the YV-repeats is not well understood. Because of the heterogeneous nature of silk fiber, it has been difficult to relate structural features to sequence. Here, we use solid-state NMR to study the conformation of valine residues in silk fiber, and in liquid silk before spinning, and we show that the valines have an identical secondary structure distribution to the alanines. This implies that the YV-repeats are part of the crystalline regions and function to demarcate their boundaries, and that the amorphous regions are formed from both AS and YV-repeats.

## Graphical abstract

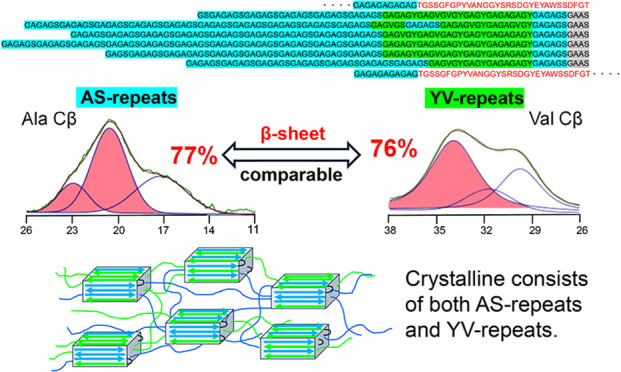

## 1 Introduction

Silk, including silkworm and spider silk, is a high-performance material that exhibits high tensile strength, moderate elasticity, biodegradability, and biocompatibility. It has attracted attention for use in biomedical applications and as a sustainable structural material.^1–5^ In the silkworm, water-soluble silk proteins (liquid silk) are stored and converted into solid fibers through mechanical stresses such as shear and tension during extrusion. This natural spinning process is of particular interest as an energy-efficient strategy for material formation.^6,7^

*Bombyx mori* silkworm silk consists of two types of proteins: fibroin, which forms the core fiber structure, and sericin, which coats the surface. Fibroin is composed of a 391 kDa heavy chain (H-chain), a 26 kDa light chain (L-chain), and a glycoprotein known as P25. The H- and L-chains are covalently linked via a disulfide bond, and P25 associates noncovalently with the H–L complex.^8–13^ The H-chain consists of 5,263 amino acid residues, composed of 45.9% Gly, 30.3% Ala, 12.1% Ser, 5.3% Tyr, 1.8% Val, and 4.7% other residues. Its primary structure includes a large central repetitive region flanked by nonrepetitive *N*- and *C*-terminal domains (Figure 1). The repetitive region, which constitutes the majority of the H-chain, is composed of Gly–X repeats, organized into 11 domains separated by linker sequences of approximately 43 residues enriched in charged amino acids.^9^ In the Gly–X repeat region, residue X is Ala in 64% of cases, Ser in 22%, Tyr in 10%, Val in 3%, and Thr in 1.3%.^8^ Within this region, the hexapeptide GAGAGS is repeated 432 times, accounting for 49% of the H-chain. In addition, sequence variants such as GAGAGY, GVGAGY, and GAGVGY—where Tyr or Val replaces Ala or Ser—are also present.^8,9^ In the remainder of this paper, we describe the hexapeptide sequences containing only G, A, and S as AS-repeats, and sequences containing Y or V as YV-repeats. These are colored blue and green, respectively, in Figure 1, which shows that each domain is made up of subdomains, each of which typically contains some AS-repeats, followed by YV-repeats, one more AS-repeat, and a 4-residue GAAS sequence.

**Figure 1:**
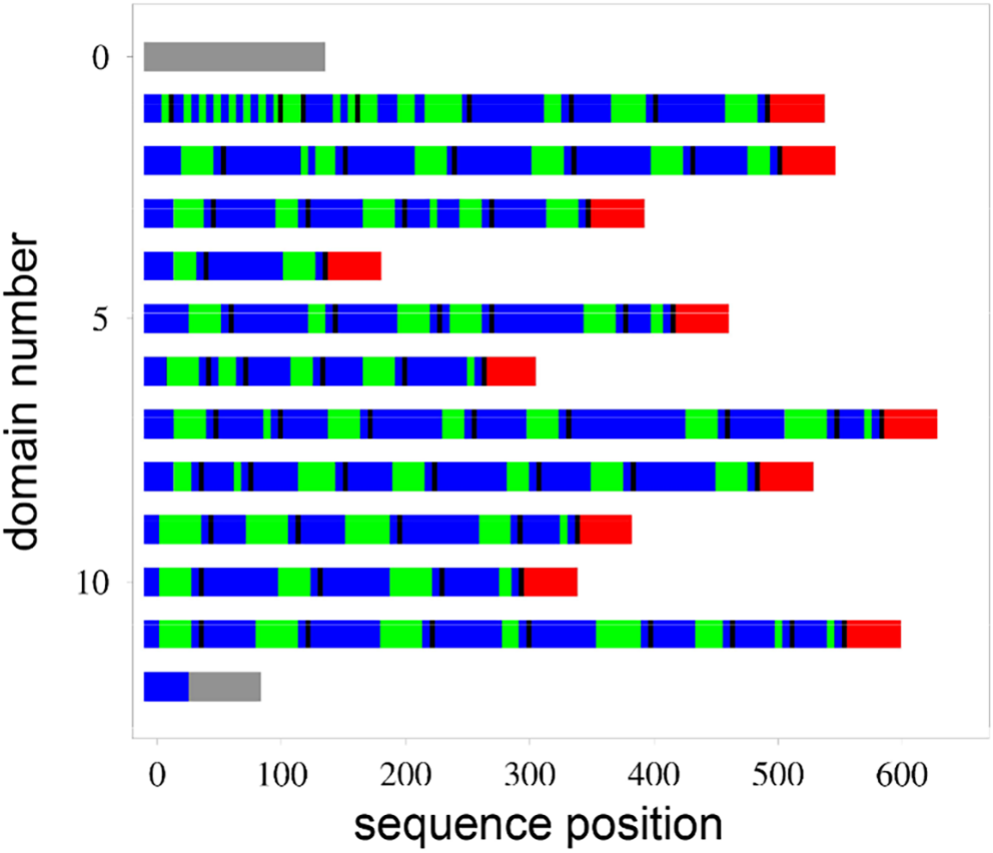
Schematic representation of the amino acid sequence of the *B. mori* silk fibroin heavy chain. The depiction of the sequence follows Zhou et al. 2001. *N*- and *C*-terminal domains are gray. The repeat sequence is divided up into 6-residue blocks. Blocks composed only of GX repeats with X = A or S (mainly GAGAGS and GAGAGA) are colored blue, while blocks containing Y or V are colored green. Each blue-green subdomain is terminated by a four-residue sequence, most commonly GAAS, in black. Subdomains are grouped into 11 domains, each of which terminates with a linker sequence of about 43 residues containing charged and aromatic amino acids in red.

Silk fibroin is stored inside the silkworm as a solution. The structure of air-dried liquid silk is referred to as Silk I, which has been shown to contain repeated type II β-turn structures, based on NMR analyses of model peptides and of liquid silk itself.^14–16^

By contrast, silk fiber contains crystalline regions, which are composed of antipolar antiparallel β-sheets, aligned with the chain direction approximately along the fiber axis, and are usually described as having Silk II structure.^17–21^ The crystallites are suggested to be about 11 nm long (ie, roughly 35 residues), 4 nm deep (roughly 8 layers), and 2.3 nm wide (roughly 6 strands side by side), although with a wide range of values.^22–24^ Treatment of silk fibers with chymotrypsin produces a crystalline fraction, which comprises about 60% of the protein,^25–27^ with the remaining 40% usually described as amorphous. NMR and other spectroscopic methods, such as IR, indicate that the β-sheet content in silk fiber is nearer 75%, presumably because about half of the “amorphous” part is locally organized into extended β-strands.

The crystalline regions must contain AS-repeats, which have been demonstrated to pack into crystalline β-sheets, with the serine hydroxyls holding strands together in a manner reminiscent of Velcro.^28^ The role of YV-repeats is less clear. The side chains of V and Y are too large to pack into a crystalline lattice. However, it has been shown by NMR that >50% of the Y residues are in β-sheet conformation, and probably >50% of V also,^17^ which could be accommodated if the Y and V sit on the surface of the crystalline regions. Tyr side chains may direct the folding of Silk I into Silk II,^29^ and are probably important for contacts between one crystalline region and another.^30^

Val is present in fibroin at an abundance even lower than that of Tyr, and its structural role and function remain largely unclear. However, this is clearly important. Takasu et al. generated genetically modified silkworms using genome editing, in which the repetitive domain of the H-chain was replaced with a highly ordered artificial sequence composed of (GAGAGS)*_n_*, (GAGAGY)*_n_*, and an amorphous region.^31^ This design excluded Val residues from the repetitive domain. As a result, the homozygous silkworms exhibited considerable degradation of fibroin in the posterior silk glands and formed cocoons composed only of sericin or did not form cocoons at all. These results strongly suggest that sequences containing Val play an essential role in fibroin secretion and fiber formation, and are likely to form part of the crystalline regions, not being merely amorphous^32^ or semicrystalline.^29,33,34^

In this study, we aimed to quantitatively characterize the structural changes before and after fiber formation in YV-repeats using solid-state NMR analysis targeting Val residues. Because Val is present in low abundance, we obtained labeled liquid silk and fiber samples suitable for efficient NMR analysis by orally administering ^13^C- and ^15^N-labeled Val to *B. mori* larvae. Solid-state NMR measurements were performed on the resulting samples, and the ^13^C, ^15^N, and ^1^H chemical shifts of Val were determined. These data revealed the secondary structure adopted by Val in the YV-repeats and the relative population of each structure. Furthermore, time-resolved MAS NMR measurements of intact liquid silk enabled us to track the structural transition from Silk I to Silk II at the Val Cβ position. We show that Val in YV-repeats behaves quantitatively the same as Ala in AS-repeats, and that both repeats compose both the crystalline and amorphous regions of silk fiber, with YV-repeats helping to define the edges of crystalline regions.

## 2 Materials and Methods

### 2.1 Sample Preparation

*B. mori* larvae (Ehime Sanshu Co., Ltd.) were fed an artificial diet (Silkmate 2S, Nosan Corporation) until day 2 of the fifth instar. From day 3 to day 5 of the fifth instar, the larvae were fed daily with 3 g of the artificial diet containing 30 mg of stable isotope-labeled Val (L-VALINE (^13^C, 99%; ^15^N, 99%), Cambridge Isotope Laboratories). After this feeding period, the larvae were fed the artificial diet until cocoon formation. Silk fibers were obtained by reeling silk from the cocoons, immersing 0.35 g of raw silk in 400 mL of 0.05% sodium carbonate aqueous solution, and heating it at 80 °C for 10 min to remove sericin. The fibers were dried overnight. The dry liquid silk sample was prepared by dissecting the posterior-middle section of the silk gland from mature larvae, washing it with distilled water to remove sericin, and drying it overnight in a Petri dish. For observation of the structural transition by MAS NMR, the posterior-middle section of the silk gland was collected from mature larvae and placed in a sample rotor.

### 2.2 Solid-State NMR Measurements

Solid-state NMR measurements of dried liquid silk and fiber samples were performed on a JEOL ECA600II spectrometer using a 3.2 mm H–X probe and an ultrafast MAS 1 mm probe. Spectral processing was conducted by using JEOL Delta software. For chemical shift referencing, the CH peak of solid adamantane was set to 29.5 ppm for ^13^C measurements, corresponding to 0 ppm relative to neat tetramethylsilane (TMS),^35^ and the peak of ^15^NH_4_Cl in its powdered form was set to 39.3 ppm for ^15^N measurements, corresponding to 0 ppm relative to liquid NH_3_.^36^ ^13^C CP-MAS NMR spectra were recorded with a contact time of 3 ms, relaxation delay of 2 s, and MAS speed of 10 kHz. The number of scans was 5000 for dried liquid silk samples and 4000 for fiber samples. ^13^C–^13^C two-dimensional DARR experiments were performed with a contact time of 3 ms, relaxation delay of 2 s, MAS speed of 20 kHz, mixing time of 0.1 s, 152 scans, and 128 increments in the indirect dimension.^37^ The acquired data were processed with Gaussian window functions (100 Hz) applied in both the t1 and t2 dimensions. ^1^H–^15^N HETCOR spectra were recorded with a contact time of 0.1 ms, relaxation delay of 2 s, and MAS speed of 14 kHz. The number of scans was 512 for liquid silk samples and 600 for fiber samples, with 32 increments in the indirect dimension. High-resolution ^1^H NMR spectra were obtained using the wPMLG pulse sequence, with MAS speed of 12 kHz and 32 scans. For wPMLG spectra, a scaling factor of 0.571 was applied, and chemical shifts were calibrated using the Ala CH_3_ peak at 1.71 ppm observed in the ^1^H ultrafast MAS spectra.^38,39^ A ^1^H ultrafast MAS solid-state NMR spectrum was acquired for the fiber sample using an ultrafast MAS 1 mm probe. ^1^H 90° pulse of 95 kHz, relaxation delay of 5 s, MAS speed of 70 kHz, and 4 scans were used. The chemical shift reference was set to 3.54 ppm, corresponding to the Gly CH_2_ peak.

The structural transition of liquid silk under MAS was measured under the following conditions. Solid-state NMR experiments were conducted by using a 400 MHz JEOL ECA400II spectrometer equipped with a JEOL 4 mm outer diameter H-X double-resonance Cryocoil MAS probe in a 9.4 T widebore magnet.^40–42^ MAS rate was 9.5 kHz at room temperature. The ^13^C DP (direct polarization)-MAS NMR spectra were recorded with an 83 kHz ^13^C 90° pulse, 93 kHz (^1^H 90° pulse) two pulse phase-modulated (TPPM) ^1^H decoupling during acquisition, and 5 s recycle delay. The ^13^C CP-MAS NMR spectra were recorded with a 1 ms constant CP pulse, 93 kHz TPPM ^1^H decoupling, and a 5 s recycle delay. In the DP- and CP-NMR measurements, 512 transients were accumulated. Signals of the mobile groups (Silk I) were obtained using the DP-MAS method, and those of the rigid groups (Silk II) were obtained using the CP-MAS method. Consecutive ^13^C DP- and CP-MAS experiments were performed alternately to monitor the structural transition of the liquid silk.

## 3 Results

### 3.1 Observation of Val ^13^C Chemical Shifts

To determine the ^13^C chemical shifts of Val residues and assign their secondary structures, ^13^C CP-MAS measurements were performed on both liquid silk and fiber samples. The ^13^C CP-MAS spectra of [^13^C/^15^N]-labeled Val in liquid silk and fiber, as well as those of nonlabeled samples, are shown in Figure 2. From the signal intensity ratio between the labeled and nonlabeled fiber spectra, the labeling efficiency of Val in the labeled sample was estimated to be 12%.

**Figure 2:**
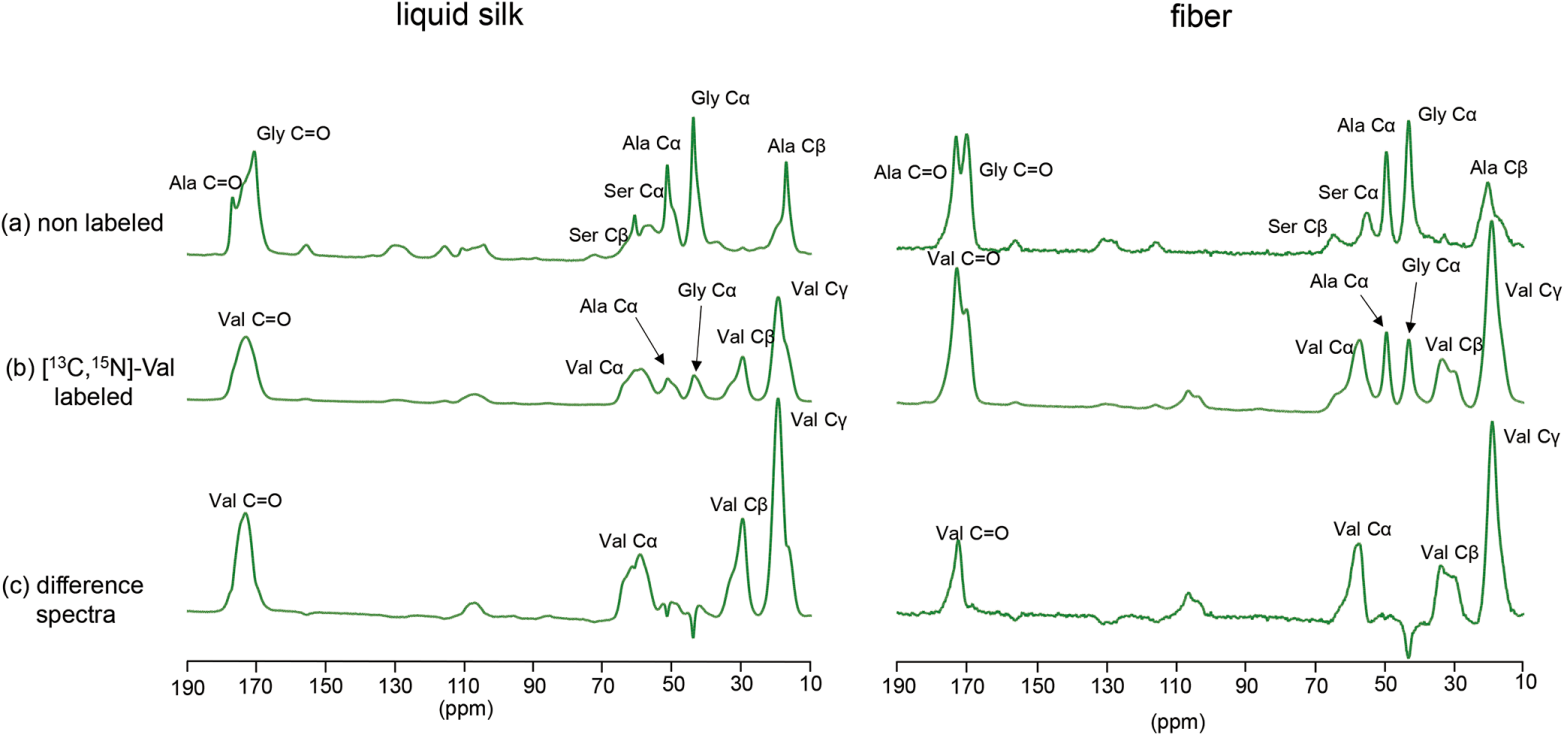
^13^C CP-MAS NMR spectra of *B. mori* silk samples highlighting valine residues. (a) Nonlabeled, (b) [^13^C,^15^N]-Val labeled, and (c) difference spectra obtained by subtracting panel a from panel b. The spectra correspond to liquid silk (left) and silk fiber (right). Characteristic peaks for Val Cα, Cβ, Cγ, and C=O carbons are observed in the labeled and difference spectra, confirming the incorporation and assignment of valine-specific signals.

Difference spectra were obtained by subtracting the nonlabeled spectra from the labeled spectra, enabling the identification of Val Cα, Cβ, Cγ, and CO signals (Figure 2). All of these peaks appeared broad and showed shoulder features, suggesting the presence of multiple structural components. Furthermore, the differences in spectral patterns between liquid silk and fiber indicated that the local structure of the Val residues differs between the two states. Notably, the Cβ peak showed a clear difference in line shape between liquid silk and fiber and did not overlap with peaks from other amino acids. Therefore, the Cβ signal was used as the primary indicator in subsequent structural analysis.

### 3.2 ^13^C–^13^C 2D DARR

Due to the presence of multiple components at the Val Cα, Cβ, and Cγ sites indicated by the ^13^C CP-MAS spectra, ^13^C–^13^C 2D DARR measurements were performed to clarify the chemical shifts of each component. The Val Cα, Cβ, and Cγ regions of the ^13^C–^13^C 2D DARR spectra for [^13^C/^15^N]-labeled Val in liquid silk and fiber are shown in Figure 3. Val possesses a continuous bonding sequence of ^13^Cα–^13^Cβ–^13^Cγ, and clear cross-peaks were observed. Although Gly and Ala are present in high abundance, they were not observed under these experimental conditions due to the low probability of direct ^13^C–^13^C bonding.

**Figure 3:**
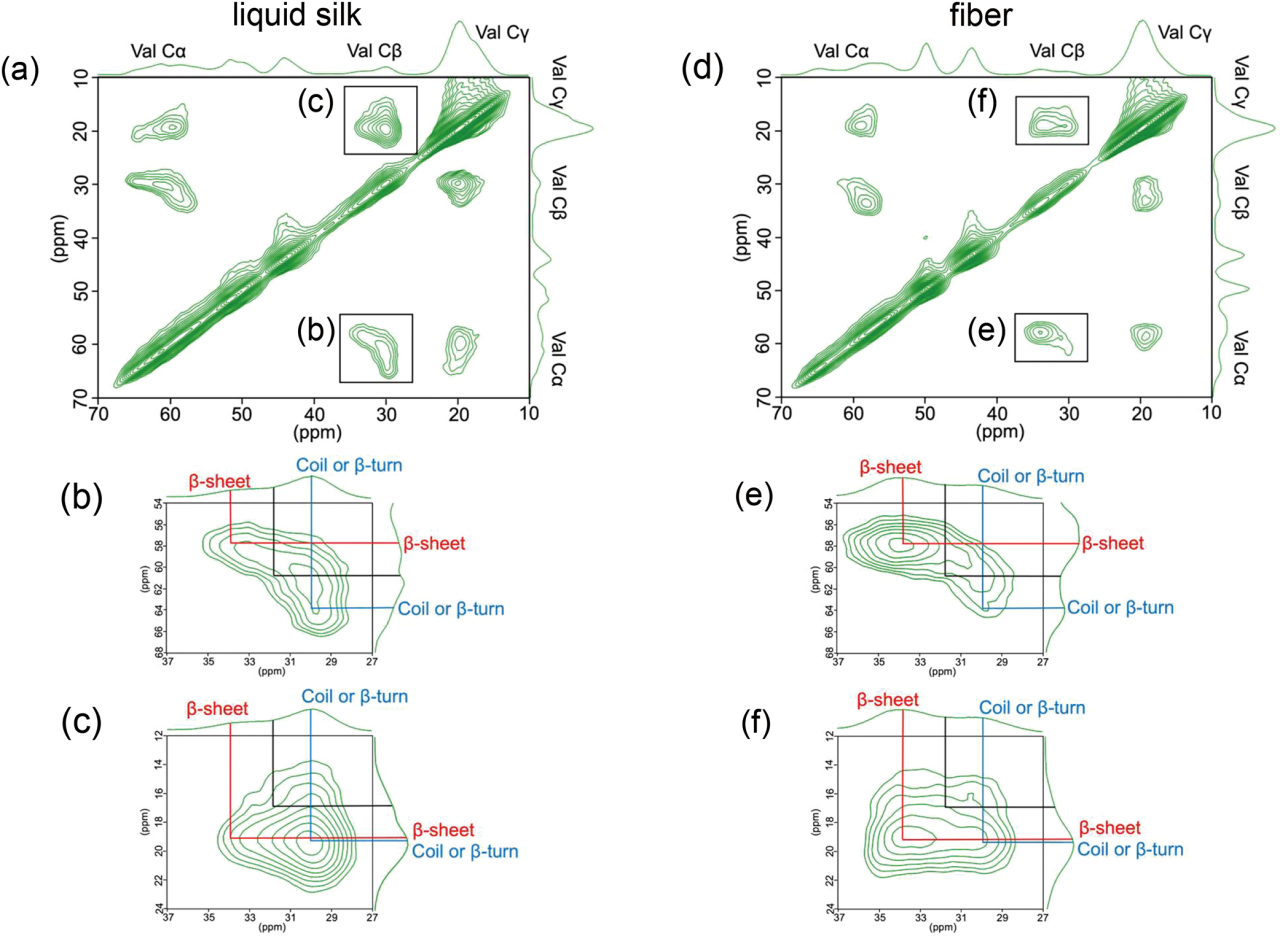
^13^C–^13^C DARR solid-state NMR spectra of [^13^C,^15^N]-Val–labeled liquid silk (a–c) and fiber (d–f). Panels b and e show enlarged views of the cross-peak between Val Cα and Cβ, and (c, f) views between Val Cβ and Cγ. These spectra confirm direct ^13^C–^13^C connectivities in valine side chains, enabling precise chemical shift assignments of individual Val carbon sites.

Cross-peaks corresponding to Val Cα–Cβ and Cβ–Cγ (Figure 3b,e,c,f, respectively) revealed that at least three components are present at the Val Cα and Cβ sites. Based on comparisons with typical secondary structure chemical shifts reported by Wishart et al. (Table S1) and previous solid-state NMR studies on fibroin structure,^43^ two of these components were assigned to β-sheet and random coil or β-turn structures. Similar approaches using broadened Val Cα–Cβ cross-peaks have been reported for disordered proteins, where the peak shape was shown to reflect the distribution of secondary structures.^44,45^ Cγ shifts (Figure 3c,f) are less useful because of signal overlap.

### 3.3 Peak Deconvolution of Val ^13^C Signals and Quantification of Structural Populations

The ^13^C–^13^C 2D DARR spectra indicate that the Val residue has at least three distinct structural components. Based on this finding, the ^13^C CP-MAS spectra for Val Cα, Cβ, and Cγ were deconvoluted into three components, and the secondary structure assignments are summarized in Table 1. The deconvolution results for Val Cβ, which does not overlap with peaks from other amino acids, are shown in Figure 4. The sum of the three fitted components reproduced the experimental spectrum well, allowing for quantification of each structural population. Spectra recorded at contact times of 1 and 3 ms were compared for dried liquid silk and fiber. As shown in Figure S1, the Val Cβ peak shapes were essentially identical, confirming that quantitative evaluation of crystalline and amorphous fractions is possible from the ^13^C CP-MAS spectra.

**Table 1:** Val ^13^C, ^15^N, ^1^HN Chemical Shifts and Structural Evaluation in Labeled Val Liquid Silk and Fiber^a^

	liquid silk		fiber		
nuclide	ppm	percentage	ppm	percentage	structure
^13^Cγ	17.0	38%	17.0	25%	
	19.5	34%	19.5	50%	
	20.7	28%	20.7	25%	
^13^Cβ	29.8	51%	29.8	30%	Coil or β-turn
	31.7	41%	31.7	15%	
	33.9	8%	33.9	55%	β-sheet
^13^Cα	57.5	ND	57.5	ND	β-sheet
	60.8	ND	60.8	ND	
	63.9	ND	63.9	ND	Coil or β-turn
^15^N	124.6	major	124.6	minor	Coil or β-turn
		minor	126.4	major	β-sheet
^1^HN	7.21	major	7.21	minor	Coil or β-turn
		minor	8.95	major	β-sheet

a ND: not determined. The Val ^13^Cα signals suffer from severe peak overlap, which prevents accurate quantitative integration.

**Figure 4:**
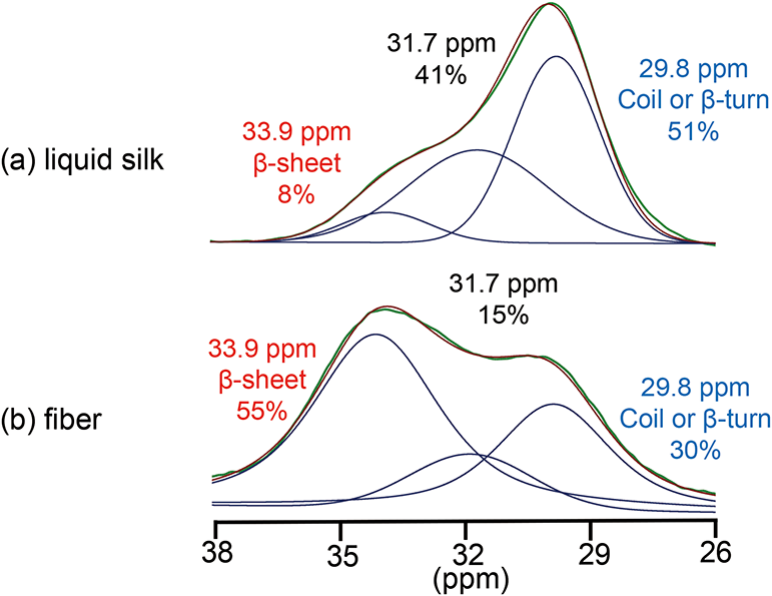
Peak deconvolution of Val Cβ in the ^13^C CP-MAS spectra of liquid silk (a) and silk fiber (b). The spectra were deconvoluted into three components based on chemical shift values obtained from ^13^C–^13^C DARR. The relative population of each component is indicated, showing increased β-sheet content upon fiber formation.

According to the literature (Table S1), the Val Cβ chemical shift values observed by solution NMR are 32.7 ppm for random coil and 33.9 ppm for β-sheet structures.^43^ By comparison of these values with our experimental data, the component at 33.9 ppm was assigned to the β-sheet structure. This assignment is supported by the observation that the β-sheet component is more abundant in the fiber sample than in liquid silk. The remaining two components were more prominent in the liquid silk than in the fiber, suggesting that they correspond to random coil or β-turn structures. Among the three components, the upfield peak at 29.8 ppm showed the largest population and the largest difference between the two samples. This peak was therefore assigned to a random coil or β-turn structure as expected for the Silk I structure. The third component, located between the β-sheet and random coil/β-turn regions, has not been assigned to a specific secondary structure at this stage. Among the Val residues present in fibroin, 42% are located outside the YV-repeats, namely, in the L-chain, linker regions, or terminal domains. It is possible that some of these residues correspond to the third component.

### 3.4 ^15^N Chemical Shifts of Val

To determine the ^15^N chemical shifts of Val, we performed ^1^H–^15^N HETCOR measurements on [^13^C/^15^N]-Val-labeled samples to achieve structural separation and assignment since one-dimensional spectra did not provide sufficient resolution. Figure S2 shows the ^1^H–^15^N cross-peaks of dried liquid silk (a) and fiber (b), and the corresponding chemical shifts are summarized in Table 1. In liquid silk, two well-defined cross-peaks were observed. Based on typical ^15^N chemical shift values of amino acids (Table S1), the high-field ^15^N peak was assigned to Gly and the low-field ^15^N peak to Val. However, in both cases, minor contributions from other residues cannot be excluded. Based on its chemical shift, the 124.6 ppm signal assigned to Val was attributed to a random coil or β-turn conformation. In contrast, the fiber sample exhibited two Val cross-peaks at 126.4 and around 125.0 ppm, which were assigned to β-sheet and random coil/β-turn structures, respectively. The peak at 124.6 ppm observed in liquid silk was also detected as a minor component of the fiber. These results indicate that Val in liquid silk predominantly adopts a random coil or β-turn structure and β-sheet structures in fiber. This conclusion is consistent with the structural assignments based on Val ^13^C chemical shifts.

### 3.5 ^1^H Chemical Shifts of Val

To determine the ^1^H chemical shifts of Val, both ultrafast MAS solid-state ^1^H NMR and high-resolution ^1^H NMR using wPMLG were conducted. The resulting chemical shift values are summarized in Table S2. The wPMLG spectra, shown in Figure S3, suggested that the HN region contains signals from multiple structural components. Based on comparison with literature values (Table S1), the peak at 7.2–7.4 ppm was assigned to random coil or β-turn structures, while the peak at 8.7–8.8 ppm was attributed to a β-sheet structure.

Figure S4 presents the ^1^HN slice spectra extracted from the ^1^H–^15^N HETCOR data. Slices corresponding to ^15^N signals at 124.6 ppm in liquid silk and 126.4 ppm in fibers were obtained. As shown in the ^1^H–^15^N correlation spectra (Figure S2), the ^1^HN chemical shifts of Val also reflected differences in secondary structure: random coil or β-turn in liquid silk and β-sheet in fiber. These results are consistent with the structural assignments based on the Val ^13^C and ^15^N chemical shifts.

### 3.6 Observation of Structural Transition from Silk I to Silk II

We previously reported that liquid silk undergoes a structural transition from Silk I to Silk II during magic angle spinning (MAS), likely due to the pressure generated within the sample rotor. We also demonstrated that this transition could be monitored over time by solid-state NMR.^46^ In this study, the same approach was applied to [^13^C/^15^N]-labeled Val samples to observe the structural transition under MAS conditions in a time-resolved manner.

Figure 5 shows the ^13^C CP-MAS and DP-MAS spectra obtained at the beginning of the measurement and after the transition process was completed. In the ^13^C DP-MAS spectrum recorded immediately after the start of measurement, the Val Cβ signal appeared as a single peak at 29.8 ppm, corresponding to the Silk I structure (random coil or β-turn). After the transition was complete, the ^13^C CP-MAS spectrum showed a decrease in the Silk I peak and the appearance of a downfield peak assigned to the Silk II structure. Although a DP experiment in principle detects all carbons, the use of a short recycle delay preferentially emphasizes mobile residues with short *T*_1_ values, thereby highlighting the Silk I component. This mobility selectivity of DP-MAS compared with CP-MAS has been demonstrated previously in fibroin^47^ and in our recent work.^46^

**Figure 5:**
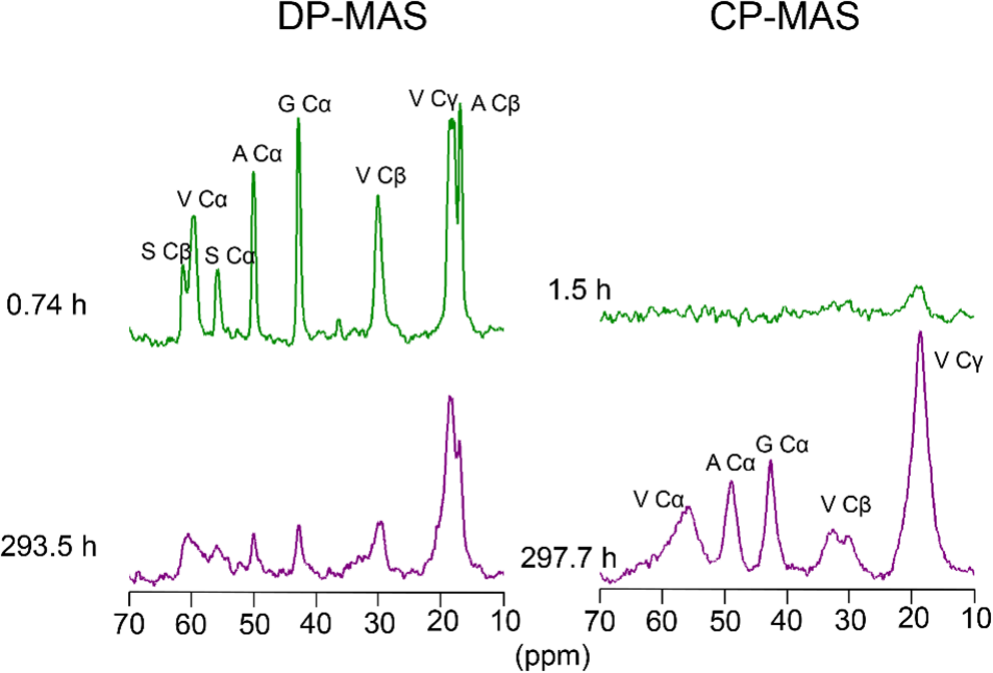
^13^C DP-MAS and CP-MAS spectra of Val–labeled liquid silk at the beginning and end of the MAS-induced structural transition. DP-MAS spectra (left) selectively detect mobile regions (Silk I), while CP-MAS spectra (right) highlight rigid regions (Silk II). Over time, the Val Cβ signal shifts downfield, indicating a transition from Silk I to Silk II.

The time course of the integrated intensities of the Silk I (from the DP-MAS spectra) and Silk II (from the CP-MAS spectra) peaks at the Val Cβ position is shown in Figure 6. The intensity of the Silk I peak began to decrease around 40 h after the start of measurement, while the Silk II peak increased concurrently. The transition continued for approximately 100 h, after which the signal intensities stabilized, indicating that the transition process had been completed.

**Figure 6:**
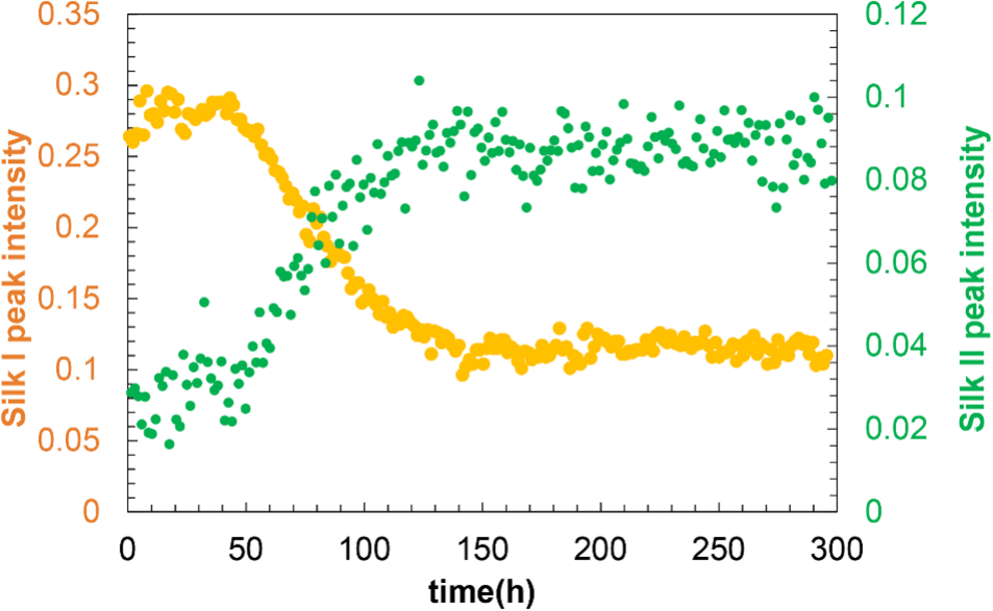
Time-dependent changes in signal intensity of Silk I (yellow) and Silk II (green) structures during the MAS-induced transition. Intensities of the Val Cβ peaks were quantified from ^13^C DP-MAS (Silk I) and CP-MAS (Silk II) spectra. A gradual decrease in Silk I and a corresponding increase in Silk II indicate a structural transition from random coil or β-turn to β-sheet conformations.

These results confirm that the Silk I structure corresponding to 29.8 ppm was transformed into the Silk II structure at 33.9 ppm under MAS-induced pressure. 30% of Silk I peaks did not transform to Silk II, indicating that the remaining signal is not due to Silk I structure. The successful time-resolved observation of this transition experimentally supports the structural assignments of Silk I and Silk II based on the ^13^C, ^15^N, and ^1^H chemical shifts of Val Cβ.

## 4 Discussion

### 4.1 Evaluation of the Secondary Structure of Val and Ala in Repeat Regions

Using [^13^C/^15^N]-labeled Val fibroin samples, we have obtained ^13^C, ^15^N, and ^1^HN chemical shifts from both liquid silk and fiber, revealing that Val residues adopt multiple secondary structures (Table 1). Since the structural assignments were consistent across ^13^C, ^15^N, and ^1^HN, we use the Val Cβ signal—least affected by peak overlap, for quantitative structural analysis.

In the fiber sample, approximately 55% of Val Cβ was attributed to the β-sheet structure (Table 1). However, Val residues are not confined to the YV-repeats; they are also found in the *N*- and *C*-terminal domains of the H-chain, the linker sequences, and the L-chain. Therefore, we used the relative distribution of Val across these regions to isolate the contribution from the YV-repeats (Table 2). Five percent of all valines are located in the C-terminal domain and 10% in the linkers. The linker is largely disordered and was classified as coil, while the C-terminal domain was classified as coil/helix based on AlphaFold predictions.^48^ In contrast, the N-terminal domain (11%) is predominantly β-sheet according to the reported crystal structure.^49^ Since 55% of all Val residues adopt β-sheet structures (Table 1) and 11% are derived from the N-terminal domain, 44% of the total Val residues (55–11) form β-sheets within the YV-repeats. Given that Val residues in the YV-repeats account for 58% of the total Val residues, the percentage of β-sheet Val within the YV-repeats is calculated as 44 × (100/58) = 76%.

**Table 2:** Val Abundance in Each Region and Their Respective Structural Proportions

region	residue	presence ratio (%)	structure	percentage of structure (%)
whole fibroin	116	100		
L-chain	19	16	coil/helix	100
N-terminal	13	11	β-sheet	100
C-terminal	6	5	coil/helix	100
Linker	11	10	coil	100
YV-repeat	67	58	β-sheet	76^a^
			coil or β-turn	24^a^

a Calculated from the observed intensity of Val Cβ at the β-sheet chemical shift (see text).

Next, to compare the structural features of Val and Ala, we evaluated the secondary structure of Ala in the repeat regions using the same region-specific approach as applied to Val (Table 3). The chemical shifts of Ala do not allow us to distinguish between Ala in AS-repeats and Ala in YV-repeats, so we first calculated the total for all GX repeats. Figure 7 shows the peak deconvolution of the Ala Cβ signal from the ^13^C CP-MAS spectrum of nonlabeled fibroin. Previous studies classified Ala residues from AS-repeats into distorted β-turns and β-sheet structures, with β-sheet structures giving two signals.^17^ As shown in Figure 7, approximately 68% of Ala Cβ was assigned to β-sheet structures. Based on this value, the secondary structure distribution of Ala residues in the AS and YV-repeats was estimated in the same manner as that for Val. Since 68% of all Ala residues form β-sheet structures and 0.7% of these are derived from the N-terminal domain, 67.3% of the total Ala residues (68 – 0.7) are β-sheet-forming Ala in the repeats. Given that Ala residues in the repeats account for 87% of the total Ala residues, the percentage of β-sheet Ala within the repeats is calculated as 67.3 × (100/87) = 77%.

**Table 3:** Ala Abundance in Each Region and Their Respective Structural Proportions

region	residue	presence ratio (%)	structure	percentage of structure (%)
whole fibroin	1630	100		
L-chain	37	2	coil/helix	100
N-terminal	12	0.7	β-sheet	100
C-terminal	6	0.3	coil/helix	100
Linker	75	5	coil	100
GAAS	82	5	coil	77
AS and YV-repeats	1418	87	β-sheet coil	23

**Figure 7:**
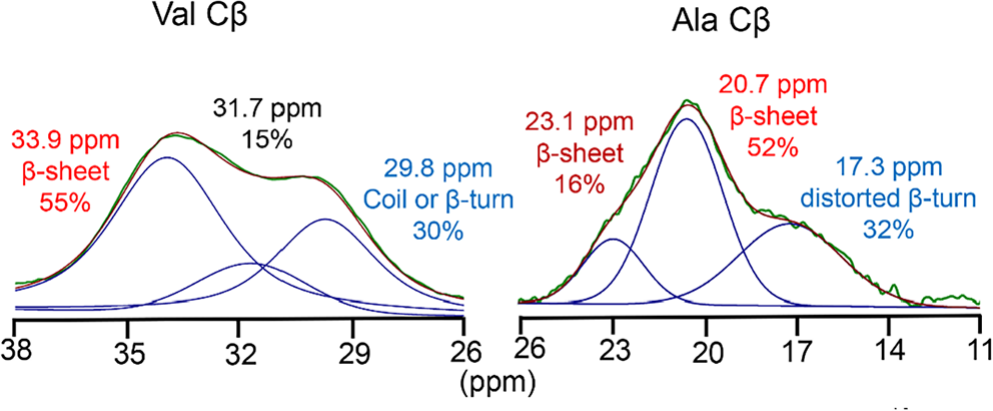
Comparison of peak deconvolution of Val Cβ and Ala Cβ in the ^13^C CP-MAS spectra of silk fiber. The Ala Cβ peak was deconvoluted into three components corresponding to β-sheet structures with different packing arrangements and distorted β-turns. The Val Cβ peak was fitted to three components assigned to the β-sheet, random coil, or β-turn, and one unassigned intermediate component.

The repeated sequences in fibroin are grouped into hexapeptides (Figure 1). It is therefore a reasonable assumption that if valines in YV-repeats are 76% β-sheet, then alanines in YV-repeats will also be approximately 76% β-sheet. The calculation above indicated that averaged overall repeats, alanine is 77% β-sheet. We therefore calculate that alanine in AS-repeats is also 77% β-sheet.

### 4.2 Comparison of the Secondary Structures of AS- and YV-Repeats

These results indicate that the β-sheet contents are identical in AS-repeats and YV-repeats. Conventionally, the (GAGAGS)*_n_* sequence has been associated with the crystalline region, whereas the YV-repeats have been variously interpreted as belonging to crystalline, semicrystalline, or amorphous regions, based very largely on the presumption that Val and Tyr side chains cannot be fitted into a crystalline packing. However, our findings quantitatively demonstrate that the proportion of β-sheet structures formed in AS and YV-repeats is the same. This result is consistent with the findings of Asakura et al.,^34,50^ who reported, based on solid-state NMR measurements using fibroin containing ^13^C- or ^15^N-labeled Tyr, Val, and Ser, that Tyr and Val residues in YV-repeats may form antiparallel β-sheet structures, similar to Ser residues in the crystalline region. The data reported in [34] show that approximately 70% of Tyr in YV-repeats is β-sheet; there is, however, a large error in this result because of chemical shift overlap, so the values for Ala, Val, and Tyr are in good agreement.

In summary, these results reveal that the proportion of β-sheet structures is identical between the AS- and YV-repeats, with both having approximately 77% β-sheet content. The repeats cover a total of 4335 residues; on this basis, the total fraction of repeat residues in crystalline β-sheet regions across heavy and light chains together should be (0.77 × 4335)/(5263 + 262) = 60%. This compares well to estimates of β-sheet content obtained experimentally,^25–27^ and implies that both AS- and YV-repeats are required to assemble together into crystallites. Specifically, this implies that YV-repeats are not amorphous or semicrystalline: they form an integral part of the crystalline regions, although presumably only at their surfaces. Such a result is consistent with the proportions of valine and tyrosine found in crystalline fractions.^27^

### 4.3 Toward a Structural Model for Silk Fibroin

Figure 1 shows the sequence of the *B. mori* heavy chain in diagrammatic form. The sequence is divided into 11 domains of variable length, each of which terminates in a roughly 43-residue linker, generally assumed to be needed for folding the structure. Each domain is made up of subdomains, each of which typically contains AS-repeats; YV-repeats; one more AS-repeat; and a 4-residue GAAS sequence, thought to form a β-turn. The AS-repeats are of variable size, while YV-repeats are less varied, with the most common length (27 occurrences out of 75 YV-repeats) being 22–26 residues long (Figure 8).

**Figure 8:**
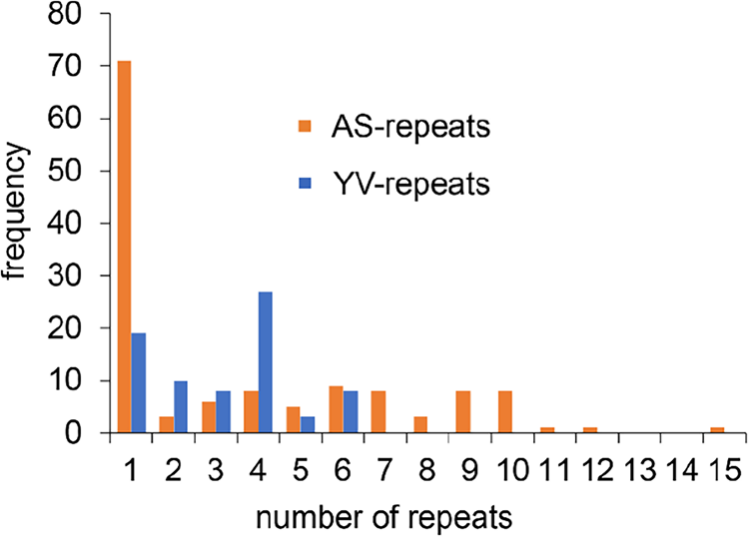
Frequency of the occurrence of hexapeptide AS-repeats and YV-repeats in the *B. mori* silk fibroin heavy chain.

This means that an alternative description of the repeat regions is that they contain typically a 26-residue YV-repeat, followed by a 6-residue AS-repeat, a GAAS turn, and a variable-length AS-repeat. Such a structure would form a hairpin approximately 32 residues long (matching the typical 11 nm length seen in crystallites) with one strand containing only GA and GS, and the other containing several Y and V. Multiple hairpins could then assemble into crystalline blocks organized in lamellar layers, keeping the YV-rich strands on the surface and AS-repeats in internal lamellar layers. In addition, another lamellar structure has also been discussed, in which antiparallel β-sheets are connected by turns every eight residues, as reported for AGAGAG and AGSGAG repeats.^30,51–53^ In this case, three or more such units assembled along the C-axis can account for the crystal C-axis length of about 11 nm. The YV-rich lamellar layer remains on the surface, and AS-repeats are located in interlamellar layers. The results reported here show that only 77% of repeats are incorporated into crystallites, with the rest presumably being unable to assemble properly and forming amorphous regions, including turns which connect β-strands. This implies that most crystallites are likely to be formed from hairpins derived from multiple polypeptide chains. The overall arrangement is indicated in Figure 9. We note that the frequent occurrence of Tyr and Val side chains on the crystallite surfaces makes the surfaces more hydrophobic and limits the size of crystalline blocks, possibly contributing to the strength and elasticity of silk fibers.^54^

**Figure 9:**
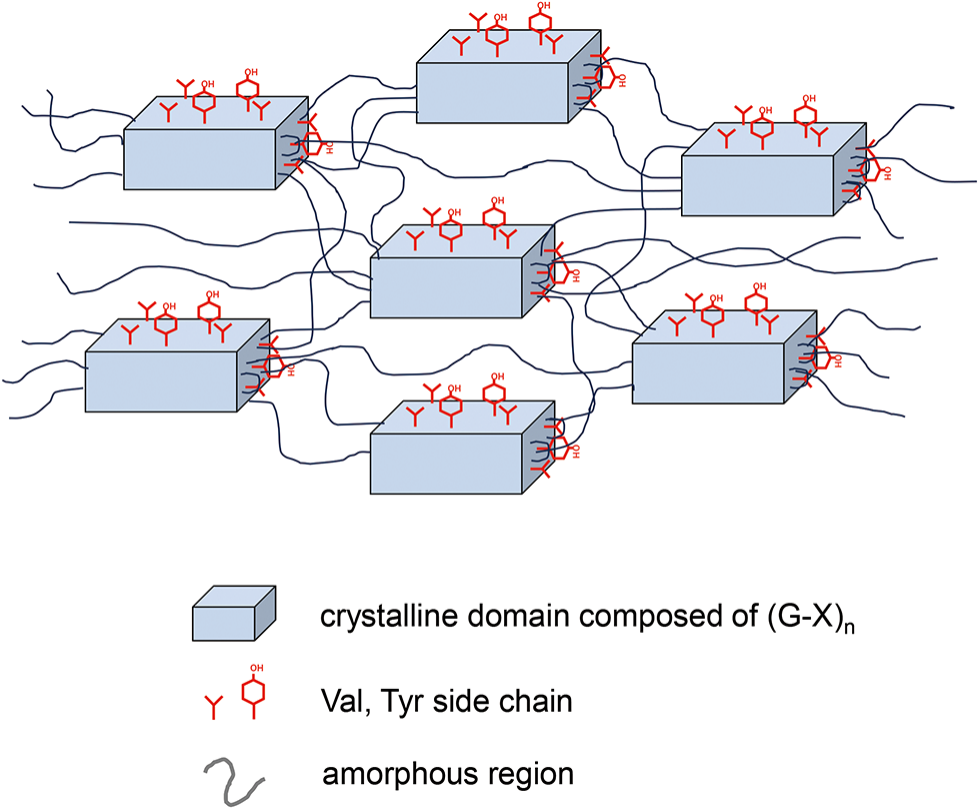
Schematic model of the structure of the silk fibroin repeated region. The crystalline region consists of stacked β-sheet structures formed by (GAGAGS)*_n_* repeats with (GAGAGY)*_n_* and (GAGVGY)*_n_* sequences on the surface. These sequences also contribute to the amorphous regions.

This structural organization reflects a hierarchical architecture similar to that of spider silk. Nova et al. proposed that the combination of highly organized β-sheet nanocrystals and extensible semiamorphous protein domains is crucial for achieving the exceptional strength and toughness of spider silk.^55^ Their mesoscale model revealed that the semiamorphous regions govern the mechanical response at small deformations by unraveling first, while the size and strength of the β-sheet nanocrystals ultimately determine the behavior at larger deformations. In particular, small β-sheet nanocrystals were shown to play a key role in enhancing both the strength and toughness. Based on these insights, we propose that in *B. mori* silk as well, the YV-repeats may control crystallite size and so prevent crystalline regions from becoming too large, thereby contributing to the mechanical properties of the fiber.

## 5 Conclusions

In this study, we used stable isotope–labeled Val fibroin samples to investigate structural changes before and after fiber formation in the YV-repeats by solid-state NMR analysis. Chemical shift data for ^13^C, ^15^N, and ^1^H showed that Val residues mainly adopt random coil or β-turn conformations in liquid silk and transform into β-sheet structures in the fiber. Deconvolution and quantification of the Val Cβ signal revealed that 76% of Val residues in the YV-repeats form β-sheet structures.

This β-sheet proportion is identical to that of Ala residues in the crystalline region, suggesting that β-sheet structures are predominant, even in the YV-repeats. Furthermore, the time-resolved observation of the MAS-induced structural transition experimentally validated the structural assignments based on the chemical shifts of Val.

Val and Tyr side chains are unable to fit into the crystalline silk packing, and we therefore suggest that they form the surfaces of crystalline regions. This creates a natural limit to the size of crystalline regions, which is useful because it is generally agreed that small crystallites are stronger.^54–58^ It also probably helps to slide crystalline domains past each other. Amorphous regions are formed from sequences (both AS- and YV-repeats) that are unable to fold into crystalline structures.

This work defines the structures and roles for most of the major structural elements in *B. mori* fibroin. Understanding and controlling the contributions of these distinct structural domains will be important for future molecular design aimed at tuning the mechanical properties of fibroin-based materials.

## Supplementary Material



## Notes

Additional solid-state NMR spectra, spectral assignments, ^13^C CP-MAS spectra of [^13^C/^15^N]­Val-labeled fibroin in the 0–80 ppm region, recorded at contact times of 1 ms and 3 ms for dried liquid silk (top) and silk fiber­(bottom); ^1^H-^15^N HETCOR spectra of liquid silk (left) and fiber (right); high-resolution ^1^H NMR spectra of liquid silk (top) and silk fiber (bottom); ^1^HN slices of Val residues extracted from the ^1^H–^15^N HETCOR spectra of liquid silk (top) and silk fiber (bottom); chemical shifts and secondary structures of each amino acid in solution NMR, adapted from ref [1]; ^1^H chemical shifts of Val-labeled liquid silk and fiber obtained by ^1^H solid-state NMR measurements (PDF)
