# Supplementary material for: Structural Analysis of Valine Residues in Silk Fibroin by Solid-State NMR
Source: J Am Chem Soc. 2025 Sep 25;147(40):36805–14. doi: 10.1021/jacs.5c13210 (PMC12512099; doi:10.1021/jacs.5c13210)
Supplement: Supplementary file 1 [file ja5c13210_si_001.pdf]

Supporting Information

Structural analysis of valine residues in silk fibroin by solid-state NMR

Misaki Mizushima[1], *Takashi Mizuno*[2], *Mitsuru Toda*[2], *Mike P. Williamson*[3], *Yu Suzuki*[1*]

[1] Department of Applied Chemistry and Biotechnology, Graduate School of Engineering, University of Fukui, 3-9-1, Bunkyo, Fukui-shi, Fukui 910-8507, Japan

[2] JEOL ltd., 3-1-2, Musashino, Akishima-shi, Tokyo 196-8558, Japan

[3] School of Biosciences, University of Sheffield, Firth Court, Western Bank, Sheffield S10 2TN, UK

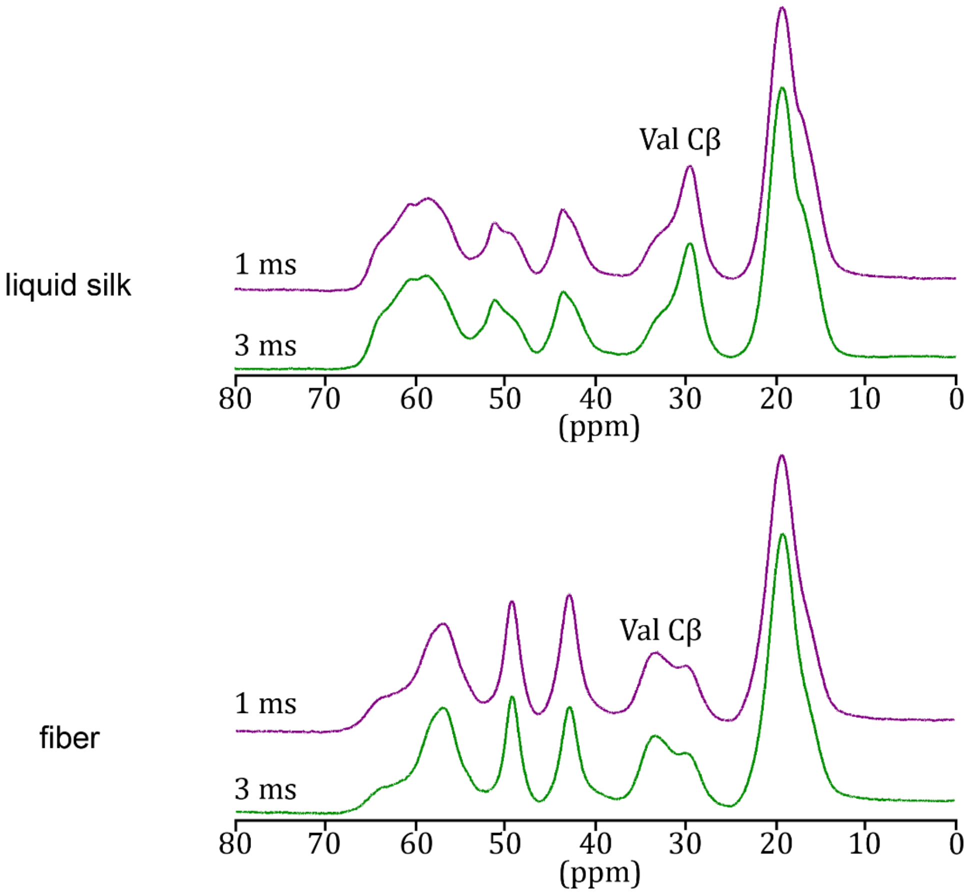

Fig S1 ^{13}C CPMAS spectra of [^{13}C/^{15}N]Val-labeled fibroin in the 0–80 ppm region, recorded at contact times of 1 ms and 3 ms for dried liquid silk (top) and silk fiber (bottom). For each sample, the upper trace shows the spectrum at 1 ms and the lower trace shows the spectrum at 3 ms. The Val Cβ peak shapes are essentially identical at the two contact times, indicating that the influence of contact time on the relative intensities is minor under our conditions.

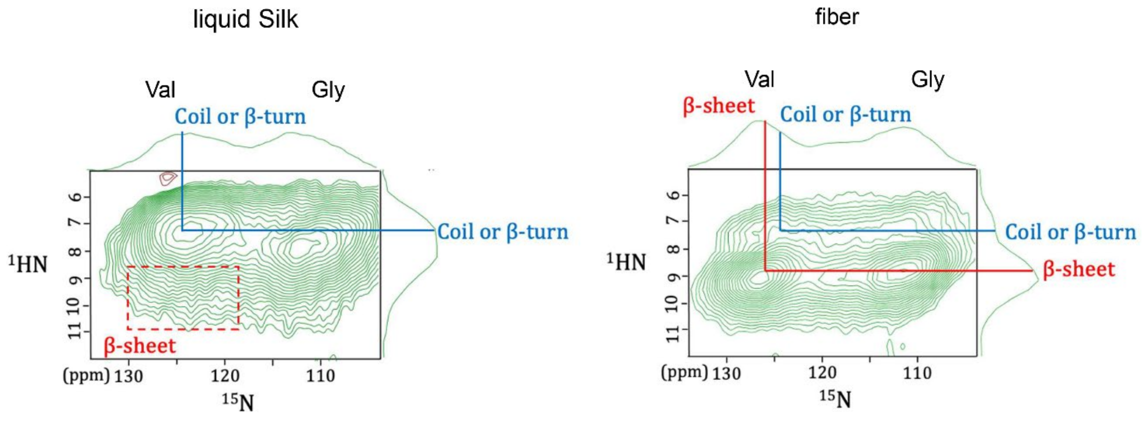

Figure S2 ^{1}H–^{15}N HETCOR spectra of [^{13}C,^{15}N]-Val–labeled fibroin in liquid silk (left) and silk fiber (right). The high-field ^{15}N signal was assigned to Gly, while the low-field signal was assigned to Val. This assignment is consistent with the typical secondary-structure-dependent ^{15}N chemical shift values (Table S1), in which Gly is located about 10 ppm upfield of Val. Cross-peaks corresponding to Val residues are observed, reflecting differences in hydrogen-bonding environments between the two states.

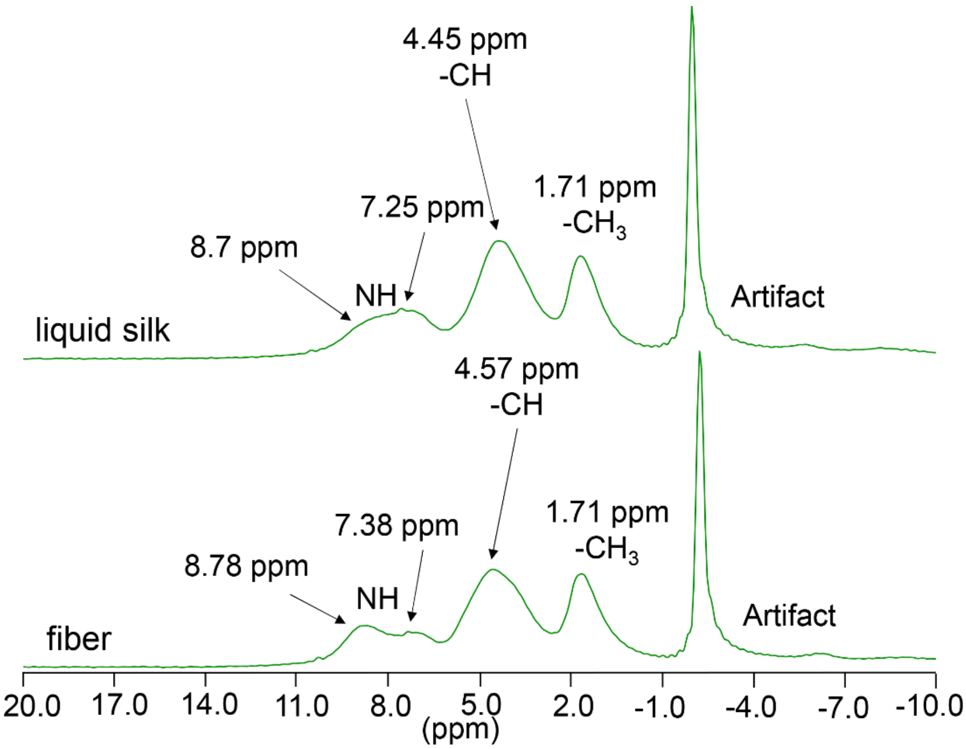

Figure S3 High-resolution ^{1}H NMR spectra of liquid silk (top) and silk fiber (bottom). Peaks assigned to Ala CH_3, CH, and Val NH groups are observed. Downfield shifts of the NH peaks in the fiber indicate the formation of β-sheet structures.

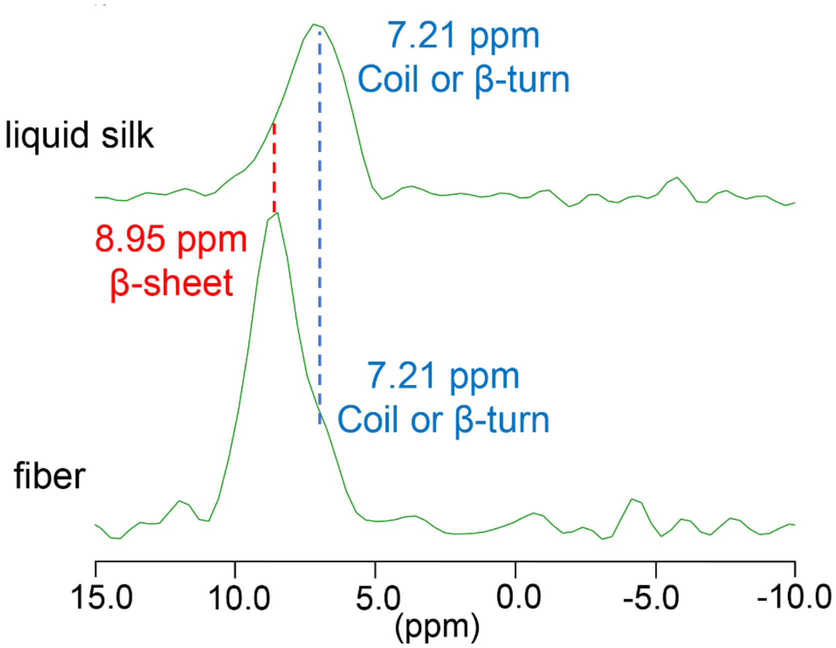

Figure S4 ^{1}HN slices of Val residues extracted from the ^{1}H–^{15}N HETCOR spectra of liquid silk (top) and silk fiber (bottom). Chemical shift values indicate that Val adopts primarily a random coil or β-turn conformation in liquid silk, whereas a β-sheet structure forms in the fiber.

Table S1. Chemical shifts and secondary structures of each amino acid in solution NMR[1]

Residue type	$^{13}C\alpha$			$^{13}C\beta$			13C'			^{15}N			^{1}HN		
	Helix	Sheet	Coil	Helix	Sheet	Coil	Helix	Sheet	Coil	Helix	Sheet	Coil	Helix	Sheet	Coil
Ala	54.8	51.5	52.8	18.3	21.1	19.1	179.4	176.1	177.7	121.4	124.5	123.6	8.08	8.44	8.15
Gly	46.9	45.2	45.5	-	-	-	175.5	172.6	173.6	107.5	109.3	109.1	8.29	8.34	8.33
Ser	60.9	57.5	58.4	63.1	65.2	64.0	175.9	173.6	174.5	114.9	116.9	115.6	8.14	8.50	8.23
Val	66.2	60.8	62.1	31.5	33.9	32.7	177.7	174.8	175.7	119.2	121.9	119.8	8.02	8.62	8.04

Table S2. ^{1}H chemical shifts of Val labeled liquid silk and fiber obtained by ^{1}H solid-state NMR measurements

	ultrafast MAS ^{1}H NMR	^{1}H wPMLG		structure
	fiber	liquid silk	fiber	
$-CH_3$	1.71	1.71	1.71	-
-CH	4.51	4.45	4.57	-
NH	7.28	7.25	7.38	Coil or β-turn
	8.78	8.7	8.78	β-sheet

Reference
[1] Wishart, D. S. Interpreting Protein Chemical Shift Data. *Prog. Nucl. Magn. Reson. Spectrosc.* 2011, 58 (1–2), 62–87. https://doi.org/10.1016/j.pnmrs.2010.07.004.
